# Al–Ca co-doped ZnO nanostructures for enhanced electrical and dielectric properties

**DOI:** 10.1038/s41598-026-61523-5

**Published:** 2026-07-24

**Authors:** Nouf Ahmed Althumairi, Imen Hammami, Sonia Soltani, H. Marzougui, R. A. El-Kasaby, Mokhtar Hjiri, Majdi Benamara, S. Soreto Teixeira, M. P. F. Graça, Manuel Almeida Valente

**Affiliations:** 1 Department of Physics, College of Science, Majmaah University, Al-Majmaah, 11952 Saudi Arabia; 2 i3N and Physics Department, University of Aveiro, Campus Universitário de Santiago, Aveiro, 3810-193 Portugal; 3 Department of Physics, College of Science, Qassim University, Buraidah, 51452 Saudi Arabia; 4 Department of Chemistry, College of Science, Taibah University, Taibah, Al-Madinah Al- Munawarah 30002, 42353 Saudi Arabia; 5 Department of Chemistry College of Science , Taibah University , Al-Madinah Al-Munawarah , 30002 Saudi Arabia; 6 Chemistry Department, Women’s College for Arts Science and Education, Ain Shams University, Cairo, 11566 Egypt; 7 Department of Physics, College of Sciences, Imam Mohammad Ibn Saud Islamic University (IMSIU), Riyadh, 11623 Saudi Arabia

**Keywords:** Al–Ca co-doped ZnO, Nanostructured semiconductor, Electrical conductivity enhancement, Dielectric and modulus analysis, Thermally activated hopping conduction, Materials science, Nanoscience and technology, Physics

## Abstract

In this work, Al (1%) and Ca (5%) co-doped ZnO (ACZO) nanopowders were successfully synthesized using a modified sol–gel method combined with supercritical ethanol drying followed by thermal annealing. X-ray diffraction confirmed that all samples crystallize in the hexagonal wurtzite structure, with a reduction of the crystallite size from 52 nm for pure ZnO to 29 nm for ACZO due to lattice distortion induced by co-doping. SEM and EDS analyses revealed a porous morphology and a homogeneous distribution of Al and Ca within the ZnO matrix. Electrical measurements showed a clear thermally activated semiconducting behaviour, with conductivity following Jonscher’s universal power law and improved charge transport in the co-doped sample compared to undoped ZnO. The activation energy extracted from DC conductivity (112 ± 5 meV) and from modulus relaxation (96 ± 5 meV) are very close, confirming that both conduction and relaxation arise from the same hopping mechanism of localized charge carriers. Impedance, Nyquist, dielectric and modulus analyses all demonstrate that Al–Ca co-doping reduces grain boundary resistance, enhances carrier mobility, and improves dielectric response. These results highlight the effectiveness of dual doping in tuning the structural and electrical properties of ZnO for potential applications in electronic, sensing, and energy devices.

**Supplementary Information:**

The online version contains supplementary material available at 10.1038/s41598-026-61523-5.

## Introduction

Nanotechnology has become a key scientific field due to its ability to tailor matter at the nanoscale and generate materials with enhanced physical, chemical, and functional properties^1,2^. In particular, nanostructured semiconductors exhibit remarkable improvements in surface area, optical absorption, electrical conductivity, and defect engineering, which make them highly suitable for advanced applications in electronics, energy conversion, sensing, and environmental remediation. Among these materials, zinc oxide (ZnO) has attracted considerable attention owing to its wide direct band gap (~ 3.37 eV), high exciton binding energy (~ 60 meV), strong piezoelectric coefficient, and excellent chemical and thermal stability^3–5^. These intrinsic properties enable ZnO to be used in a broad range of applications such as optoelectronic devices, gas sensors, photocatalysis, solar cells, spintronics, and transparent conductive oxides (TCOs). However, the practical performance of ZnO is often limited by its moderate electrical conductivity and fast charge recombination, which restrict its efficiency in electronic and energy-related applications^6–8^.

Doping engineering has proven to be one of the most effective strategies to enhance and tune the physical properties of ZnO. In particular, aluminium (Al) doping is widely used to obtain n-type ZnO with improved electrical conductivity and high optical transparency, making it a promising alternative to conventional TCOs such as indium tin oxide (ITO)^9,10^. Nevertheless, Al-doped ZnO often suffers from thermal and environmental instability, as well as limited control over defect chemistry at higher doping levels. On the other hand, calcium (Ca) doping in ZnO has been reported to significantly influence the structural, optical, and dielectric properties of the material^11,12^. Due to its larger ionic radius compared to Zn²⁺, Ca incorporation induces lattice distortion, modifies defect states, and can reduce exciton recombination rates. Furthermore, Ca doping has been shown to enhance photocatalytic activity, dielectric response, and optical band gap tuning, which are essential for optoelectronic and sensing applications^13,14^.

The combination of a donor dopant (Al³⁺), which increases free carrier concentration, with a modifying dopant (Ca²⁺), which induces lattice distortion and tailors defect chemistry, is expected to offer an effective strategy to simultaneously enhance electrical conductivity, dielectric response, and structural stability^15,16^. In addition, co-doping can improve dopant solubility, minimize defect clustering, and enhance long-term material stability compared to single-doped systems. In this context, the present work focuses on the design and investigation of Al–Ca co-doped ZnO nanoparticles as a multifunctional material with improved structural, electrical, and dielectric properties. The co-doped ZnO powders were synthesized using a cost-effective and scalable chemical route, and their structural, morphological, and dielectric characteristics were systematically analyzed to elucidate the role of dual dopant incorporation^17–19^. This study provides deeper insight into defect engineering and charge transport mechanisms in co-doped ZnO systems and highlights the potential of Al–Ca co-doped ZnO for applications in transparent electronics, sensors, and energy-related devices.

## Experimental procedure

### Synthesis

Pure ZnO and aluminum-calcium co-doped ZnO nanopowders were prepared using a modified sol–gel method. For the synthesis of undoped ZnO, 16 g of zinc acetate dihydrate [Zn(CH₃COO)₂·2 H₂O] was dissolved in 112 mL of methanol and stirred magnetically at room temperature for 10 min until a homogeneous solution was obtained. For the co-doped ZnO samples (ACZO), the same initial solution was prepared using zinc acetate dihydrate in methanol. After 10 min of stirring, an appropriate amount of aluminum nitrate nonahydrate [Al(NO₃)₃·9 H₂O] was added to obtain an atomic ratio [Al]/[Zn] = 0.01, followed by the addition of calcium chloride hexahydrate [CaCl₂·6 H₂O] to reach [Ca]/[Zn] = 0.05. The mixture was then stirred for an additional 15 min to ensure good homogenization and uniform distribution of Al³⁺ and Ca²⁺ ions in the precursor solution.

For both pure and co-doped solutions, the obtained sol was then transferred into an autoclave and subjected to supercritical drying under ethanol conditions (critical temperature T_c_ = 243 °C and critical pressure P_c_ = 63.6 bar). This step allowed the formation of dry nanopowders with a highly porous and homogeneous structure. Finally, all synthesized samples were annealed in air at 400 °C for 2 h in order to improve their crystallinity and phase purity. For comparison, reference samples doped individually with aluminum or calcium and other compositions were also prepared using the same procedure. In the following, the aluminum and calcium co-doped ZnO nanopowders are denoted as ACZO.

### Structural and morphological characterization

The X-ray diffraction (XRD) patterns were recorded at room temperature using an Aeris PANalytical diffractometer with CuKα radiation (λ = 1.54056 Å). The data were collected over a 2θ range from 30° to 70° with a step size of 0.02°.

The morphology of the prepared powders was examined using Scanning Electron Microscopy (SEM) with a TESCAN Vega 3 instrument. The chemical composition was analyzed in a semi-quantitative way using an Energy Dispersive Spectroscopy (EDS) system (Bruker QUANTAX) attached to the SEM. A focused electron beam with an approximate spot size of 5 μm was used to analyze selected regions of the sample surface.

### Electrical characterization

Electrical measurements were carried out on pellet samples with a thickness of about 1 mm and a diameter of 7 mm. Impedance measurements were performed using an Agilent 4294 A impedance analyzer in the frequency range from 100 Hz to 1 MHz, using the Cp–Rp configuration and an applied AC voltage of 0.5 V.

The measurements were performed in a nitrogen cryostat, allowing temperature-dependent analysis in the range of 180–360 K. The temperature was controlled using an Oxford Research IT-C4 controller, and the sample temperature was monitored using a calibrated platinum sensor.

## **Results and discussions**

### Structural characterizations

**Fig. 1:**
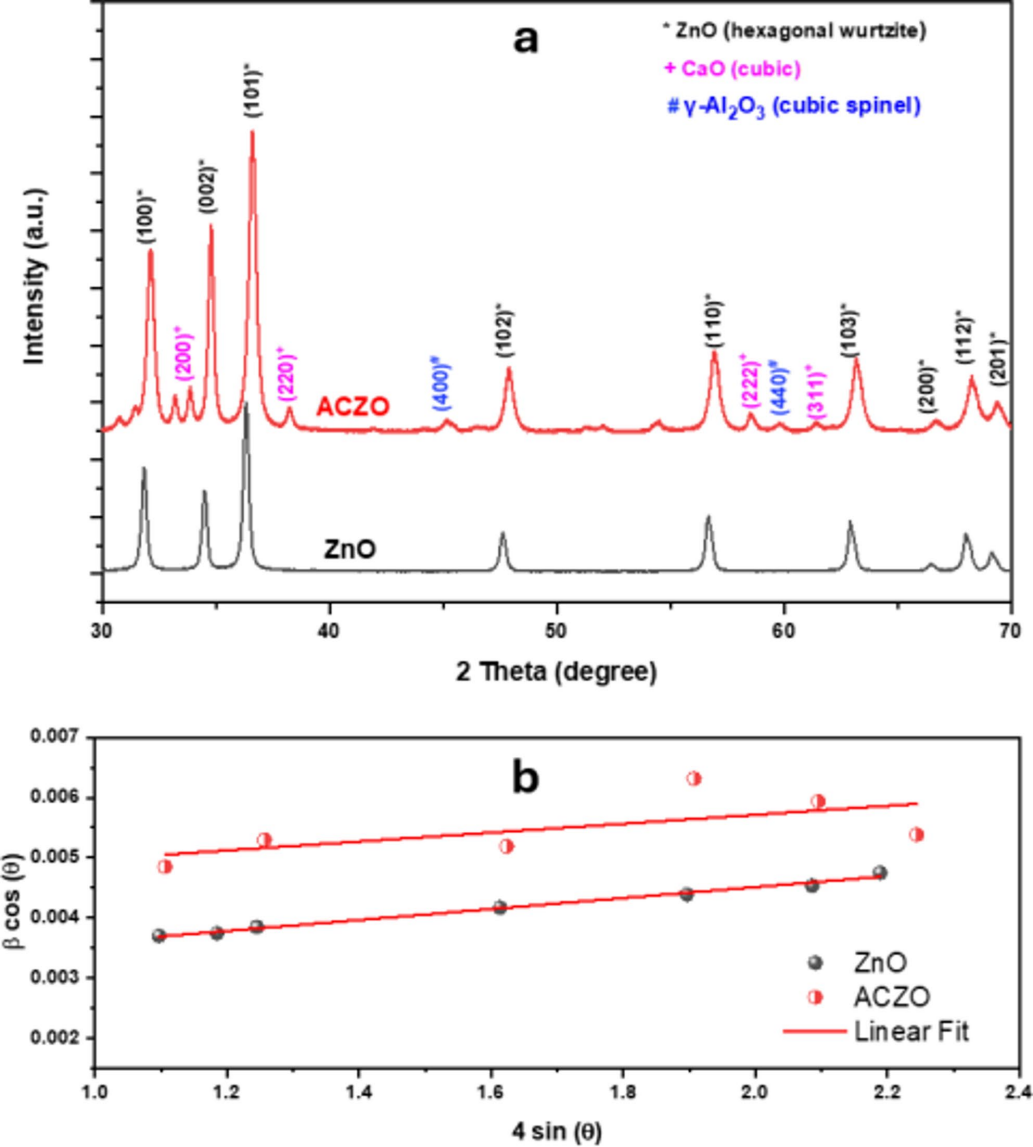
XRD patterns of the ZnO and ACZO (Al 1% + Ca 5% co-doped ZnO) powders.

The XRD patterns of pure ZnO and ACZO (Al 1% + Ca 5% co-doped ZnO) shown in Fig. 1**(a)** confirm that the hexagonal wurtzite structure of ZnO is preserved after co-doping, with all the principal reflections indexed to the (100), (002), (101), (102), (110), (103), (200), (112), and (201) planes^20,21^. The absence of additional major peaks in the main pattern and the preservation of peak positions indicate that the fundamental crystal structure remains largely unchanged, although a slight broadening of the diffraction peaks is observed for the ACZO sample, suggesting the introduction of lattice distortion, defects, and microstrain induced by the incorporation of Al³⁺ and Ca²⁺ ions. The crystallite size was initially estimated using the Scherrer equation applied to the most intense (101) reflection. The undoped ZnO sample exhibits an average crystallite size of approximately 52 nm, indicating well-developed crystalline domains, whereas the ACZO sample shows a reduced crystallite size of about 29 nm, revealing significant grain refinement after co-doping. This reduction in crystallite size can be attributed to lattice strain and defect generation caused by the difference in ionic radii between Zn²⁺, Al³⁺, and Ca²⁺, which inhibits crystal growth and promotes nucleation of smaller crystallites^22,23^.

To quantitatively evaluate the strain induced by doping, Williamson–Hall (W–H) analysis was performed using the uniform deformation model, according to the relation $\:\beta\:\mathrm{cos}\theta\:=\frac{k\lambda\:}{D}+4\epsilon\:\:\mathrm{s}\mathrm{i}\mathrm{n}\theta\:$, and the corresponding linear fits are presented in Fig. 1**(b)**. From the W–H fitting, the crystallite size of pure ZnO was determined to be 52 nm with a lattice strain of $\:9.11\times\:{10}^{-4}$. For the ACZO sample, the crystallite size decreased to 33 nm, while the strain was calculated as $\:7.41\times\:{10}^{-4}$. The positive strain values indicate an overall tensile strain in both samples. The decrease in crystallite size after co-doping is consistent with enhanced defect formation and inhibited crystal growth.

The reduced peak intensity and broadening in ACZO indicate lower crystallinity, smaller crystallite size, and slight preferred orientation changes caused by dopant-induced lattice distortion^24,25^.

In addition to the main ZnO reflections, the ACZO pattern exhibits several weak secondary peaks, confirming the formation of minor secondary phases due to dopant segregation at higher Ca content. The peaks marked with (+) in Fig. 1**(a)** are assigned to cubic CaO (space group Fm3̅m), while those marked with (#) correspond to γ-Al₂O₃ with a cubic spinel structure (space group Fd3̅m). These phases arise from the limited solubility of Ca in ZnO and local segregation of Al, respectively. Some reflections show partial overlap between CaO and γ-Al₂O₃, particularly in the 2θ ranges around 38°, 45°, and 60–61°, due to their similar cubic structures and close interplanar spacings. Overall, the XRD results demonstrate that the ACZO sample remains predominantly composed of the wurtzite ZnO phase, with only trace amounts of CaO and γ-Al₂O₃ secondary phases.

The presence of weak CaO and γ-Al₂O₃ reflections indicates that a small fraction of the dopants remains segregated outside the ZnO lattice. Although these secondary phases are present only in trace amounts, they may influence the microstructural and electrical behavior of the material. In particular, CaO and γ-Al₂O₃ are expected to be preferentially located at grain boundaries, where they can modify local potential barriers and interfacial polarization processes. Such grain boundary modifications may contribute to the observed dielectric response and charge transport behavior. However, considering the low intensity of their diffraction peaks and the dominance of the ZnO wurtzite phase, the overall electrical and dielectric properties are mainly governed by the co-doped ZnO matrix, while the secondary phases act as a secondary contribution through grain boundary effects.

A closer inspection of the diffraction pattern reveals weak shoulder features around 2θ ≈ 32° and near 55°. The shoulders located on both sides of the ZnO (100) reflection are attributed mainly to lattice distortion, microstrain, and local structural disorder induced by the simultaneous incorporation of Al³⁺ and Ca²⁺ ions into the ZnO lattice^11,26^. The large difference between the ionic radii of Zn²⁺ (0.74 Å), Al³⁺ (0.53 Å), and Ca²⁺ (1.00 Å) generates local lattice distortions that can produce peak asymmetry and broadening around the main ZnO reflections. The weak feature observed near 55° can be associated with the low-intensity (220) reflection of cubic CaO, supporting the presence of a minor CaO secondary phase^27^.

Although Al³⁺ substitution locally tends to induce compressive stress because of its smaller ionic radius, whereas Ca²⁺ favors local lattice expansion due to its larger ionic radius, the Williamson–Hall analysis reveals that the net residual strain remains tensile in both samples, with pure ZnO showing a strain of $\:9.11\times\:{10}^{-4}$and ACZO showing $\:7.41\times\:{10}^{-4}$. The coexistence of these dopants therefore creates competing local compressive and tensile stress fields, leading to structural disorder, defect formation, and grain boundary modification rather than a purely compressive or purely tensile global lattice response. This effect is evidenced by peak broadening, slight peak asymmetry, and reduced crystallite size in the ACZO sample, which subsequently influence the electrical and dielectric behavior.

#### Morphological and chemical composition of the Al-Ca co-doped ZnO (ACZO)

**Fig. 2:**
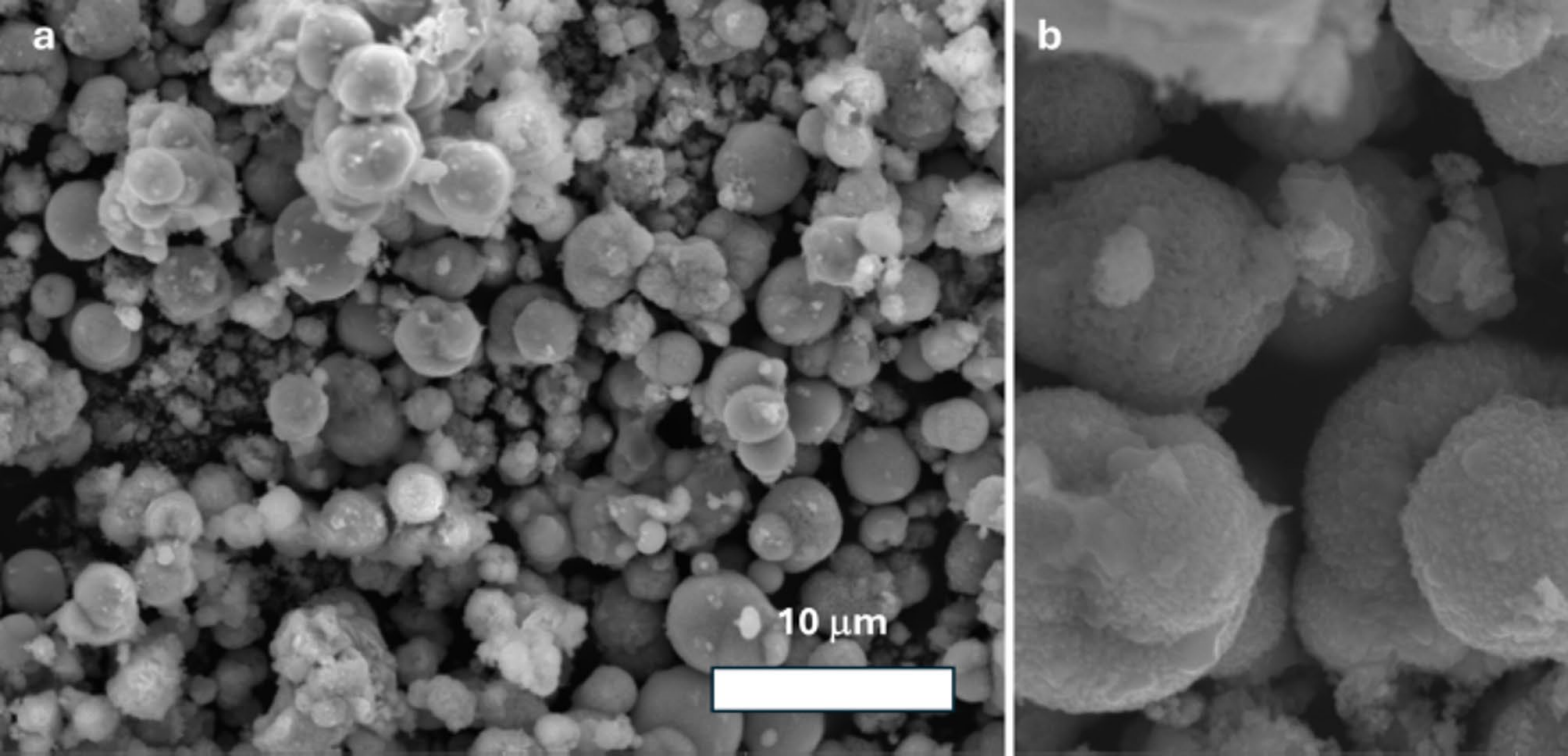
SEM images of the ACZO in two scales (**a**) 10 μm and (**b**) 2 μm.

The SEM images provide a clear observation of the morphology of the synthesized powders at two different magnifications. In Fig. 2-a, recorded at low magnification (10 μm scale), the sample appears as a highly agglomerated assembly of particles forming a porous network. The particles exhibit mainly quasi-spherical shapes with a wide size distribution, ranging from submicron grains to larger micrometric ones. This type of agglomeration is commonly observed in ZnO nanopowders prepared by wet chemical routes and supercritical drying, due to the high surface energy that promotes particle–particle adhesion. In Fig. 2-b, at higher magnification (2 μm scale), each large spherical particle is clearly composed of many smaller primary grains. The particle surfaces appear rough and granular, indicating that the spheres are formed by the aggregation of nanocrystallites. The observed agglomerated polycrystalline morphology suggests the presence of numerous grain boundaries. Although SEM alone cannot directly confirm their electrical role, the impedance and Nyquist results support that grain boundaries significantly contribute to charge transport and dielectric polarization.

**Fig. 3:**
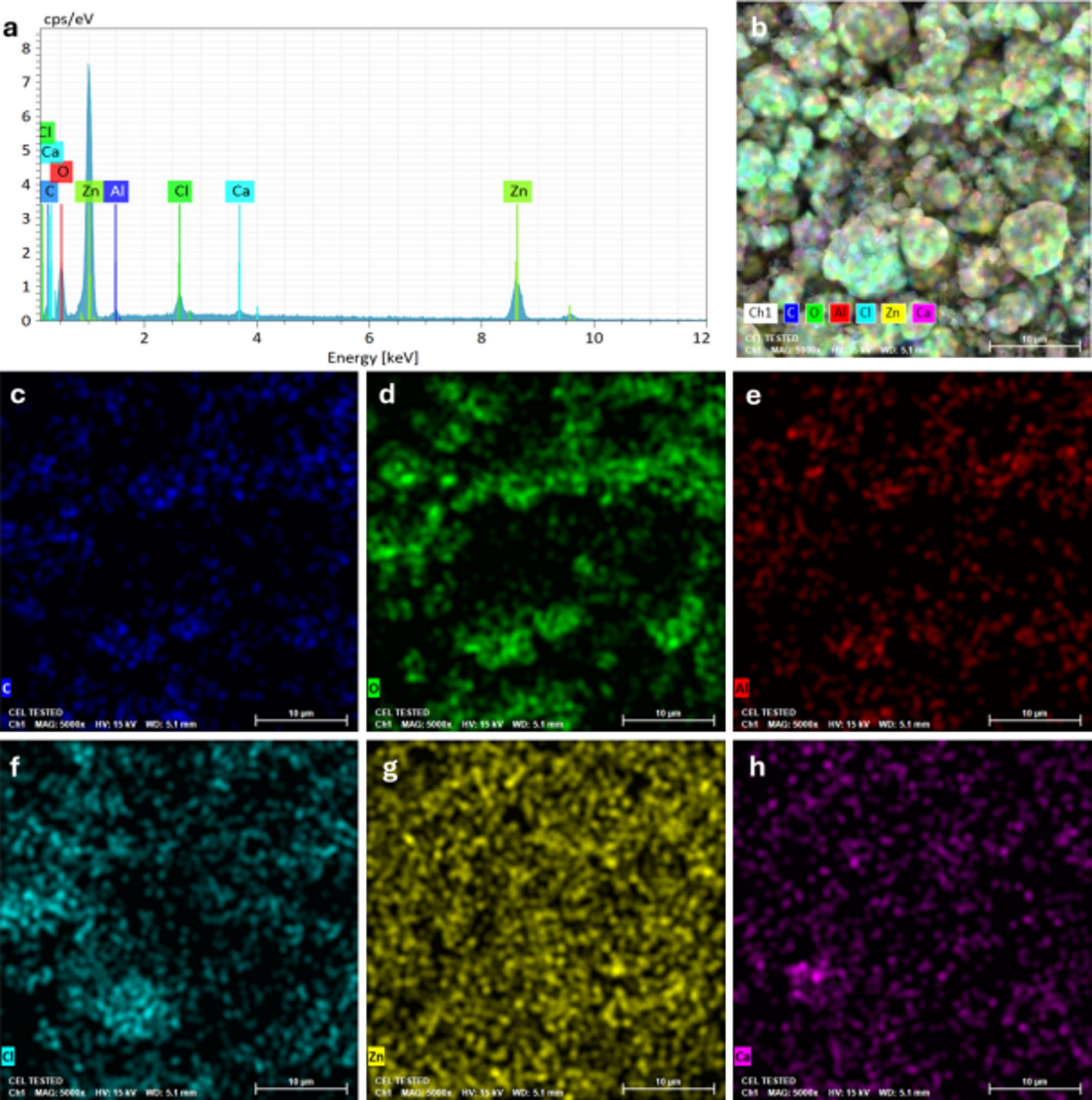
(**a**) EDS spectrum confirming the presence of Zn, O, Al and Ca in the ACZO nanopowders, (**b**) corresponding SEM image of the mapped area, and (**c–h**) elemental mapping images showing the distribution of (**c**) C, (**d**) O, (**e**) Al, (**f**) Cl, (**g**) Zn and (**h**) Ca, indicating a quite uniform dispersion of all elements in the sample.

In order to verify the chemical composition and homogeneity of the ACZO nanopowders, the elemental mapping and EDS analysis were presented in Fig. 3. In Fig. 3-a, the EDS spectrum clearly shows the main characteristic peaks of Zn and O, which verify the formation of the ZnO host structure. In addition, the presence of Al and Ca peaks confirms the successful introduction of both dopant elements into the synthesized powder. However, EDX alone cannot distinguish between substitutional lattice incorporation and segregated dopant-rich phases. A small Cl signal is also detected, which can be attributed to residual traces from the calcium precursor (CaCl₂·6 H₂O). No additional impurity peaks are observed, indicating a good chemical purity of the synthesized powders.

Figure 3-b shows the elemental mapping of the selected area, where all detected elements (C, O, Al, Cl, Zn and Ca) are visualized together. The contrast appears quite homogeneous over the whole region, suggesting a uniform dispersion of the elements within the powder. The individual elemental maps in Fig. 3-c–h provide more detailed information. The carbon signal in Fig. 3-c mainly comes from the conductive carbon tape used during SEM observation. The oxygen (Fig. 3-d) and zinc (Fig. 3-g) maps show a continuous and uniform distribution, confirming the formation of a homogeneous ZnO matrix. The aluminium (Fig. 3-e) and calcium (Fig. 3-h) maps indicate that both dopants are well distributed in the sample without visible segregation. The chlorine map (Fig. 3-f) shows a weak but dispersed signal, consistent with a small residual amount from the precursor. EDX and XRD provide complementary information. EDX confirms the presence and homogeneous distribution of Al and Ca, whereas XRD reveals that a small fraction of dopants segregates as CaO and γ-Al₂O₃ secondary phases. This indicates partial lattice incorporation together with minor dopant segregation.

#### Dielectric characterization

**Fig. 4:**
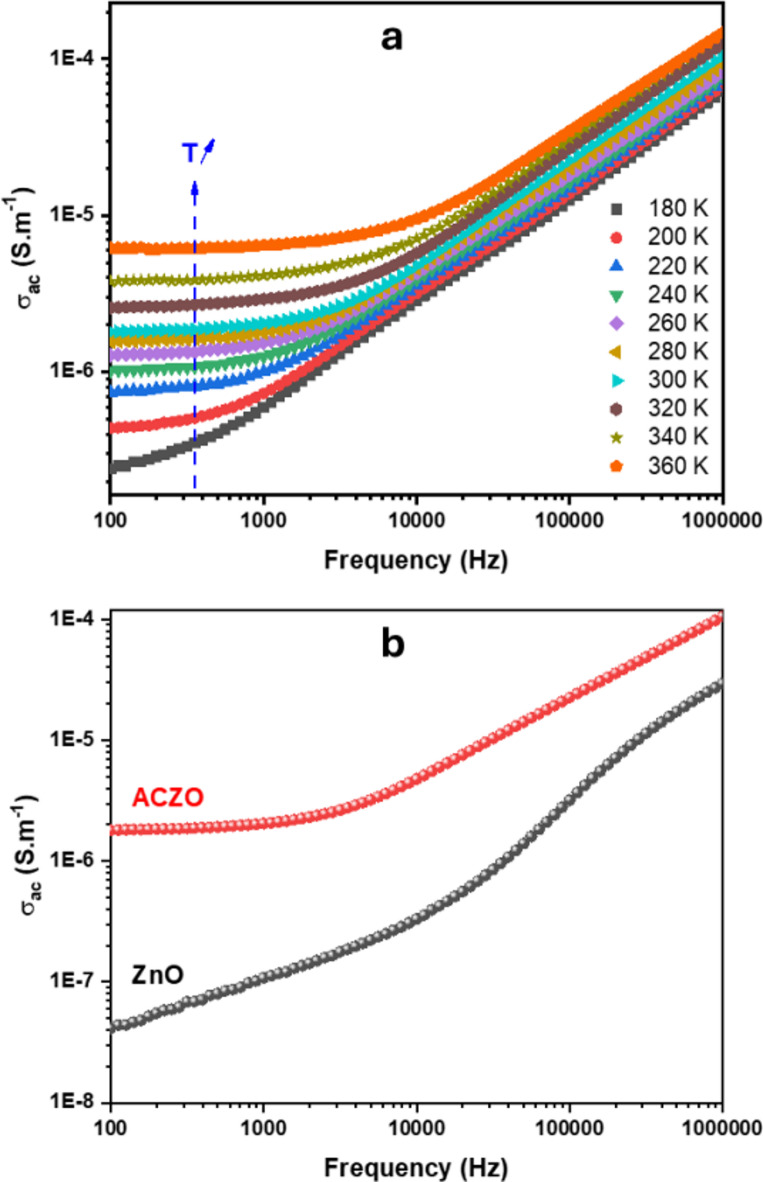
(**a**) *shows the AC electrical conductivity vs. frequency at different temperatures of the ACZO sample (Al 1%*,* Ca 5% co-doped ZnO)*,* and* (**b**) *comparison of the AC electrical conductivity vs. frequency at 300 K for ACZO and pure ZnO*.

The electrical conductivity of the ACZO sample in Fig. 4-a shows a clear semiconducting behaviour with a strong dependence on temperature. The conductivity increases gradually with increasing temperature, which indicates a thermally activated transport mechanism. At low temperatures, charge carriers are more localized and partly trapped at defects and grain boundaries, leading to low conductivity. When the temperature increases, the carriers gain sufficient thermal energy to overcome these barriers, and the electrical conduction becomes easier. The frequency response follows the universal Jonscher power law, σ(ω) = σ_dc_ + Aωⁿ^28^. The AC conductivity analysis reveals two distinct regions: a low-frequency plateau corresponding to DC conductivity and a high-frequency dispersive region associated with localized carrier hopping. The increase in conductivity with both frequency and temperature confirms a thermally activated conduction mechanism consistent with Jonscher’s universal power law. A plateau is observed at low frequency corresponding to the DC conductivity (long-range transport), while at higher frequencies the conductivity increases due to hopping of localized carriers between defect states. This behaviour suggests that the conduction process in ACZO is mainly governed by thermally activated hopping and grain boundary effects, which is typical for polycrystalline ZnO-based materials^29,30^. Trace CaO and γ-Al₂O₃ may additionally create localized trapping centers that slightly affect carrier hopping.

The co-doping effect of Al and Ca plays an important role in improving the electrical transport. The substitution of Zn²⁺ by Al³⁺ introduces additional free electrons and increases the carrier concentration, while Ca²⁺ modifies the defect structure and helps to reduce potential barriers at the grain boundaries, which facilitates carrier mobility. As a result, the ACZO sample exhibits better electrical conduction than pure ZnO. This is confirmed in Fig. 4-b, where the conductivity at 300 K of ACZO is higher than that of undoped ZnO. These results indicate that Al–Ca co-doping is an effective strategy to enhance the electrical properties of ZnO, making this material more suitable for applications such as sensors and electronic devices where improved charge transport is required^17,31^.

**Fig. 5:**
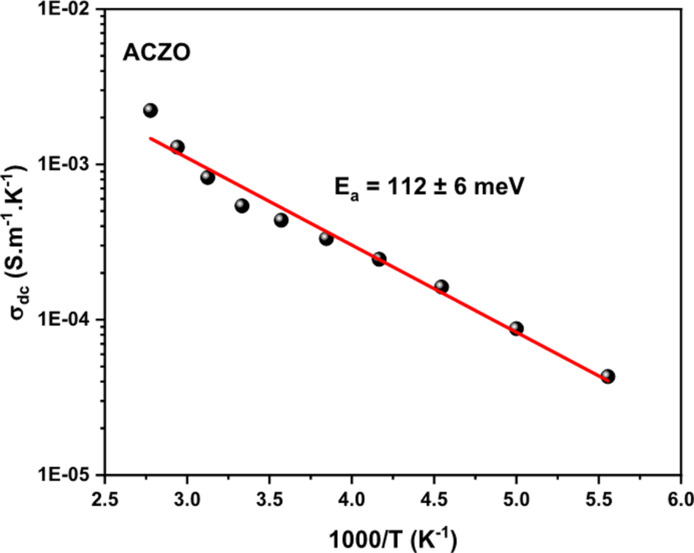
Arrhenius plot of the ACZO sample.

Figure 5 displays the DC electrical conductivity change as a function of 1000/T. The Arrhenius representation clearly demonstrates a linear pattern, confirming that the conduction mechanism in the ACZO sample is thermally triggered. Plotting ln(σ_dc_) against 1000/T yields a straight line with a slope of -E_a_/k_B_ because the DC conductivity in this kind of plot follows the formula σ_dc_ = σ_0_ exp(-E_a_/k_B_T). Direct extraction of the activation energy E_a_ from the slope is possible by linear fitting of the experimental data. The very low value (112 ± 5 meV) observed suggests that hopping mechanisms of charge carriers between localized states, rather than band conduction, drive electrical transport. This phenomenon is common for doped ZnO nanostructures, where localized energy levels are created inside the band gap by defects such zinc interstitials, oxygen vacancies, and dopant-related states. Al³⁺ dopant raises the concentration of free electrons, while Ca²⁺ alters the defect landscape and grain boundary potential barriers, all of which lower the energy needed for carrier migration. As a result, the energy barrier that carriers must cross in order to travel across grain boundaries or from one localized location to another is represented by the extracted activation energy. In good accord with the conductivity boost seen at higher temperatures, the comparatively modest value of E_a_ validates that the co-doping approach promotes charge transfer and enhances electrical conductivity^32,33^.

**Fig. 6:**
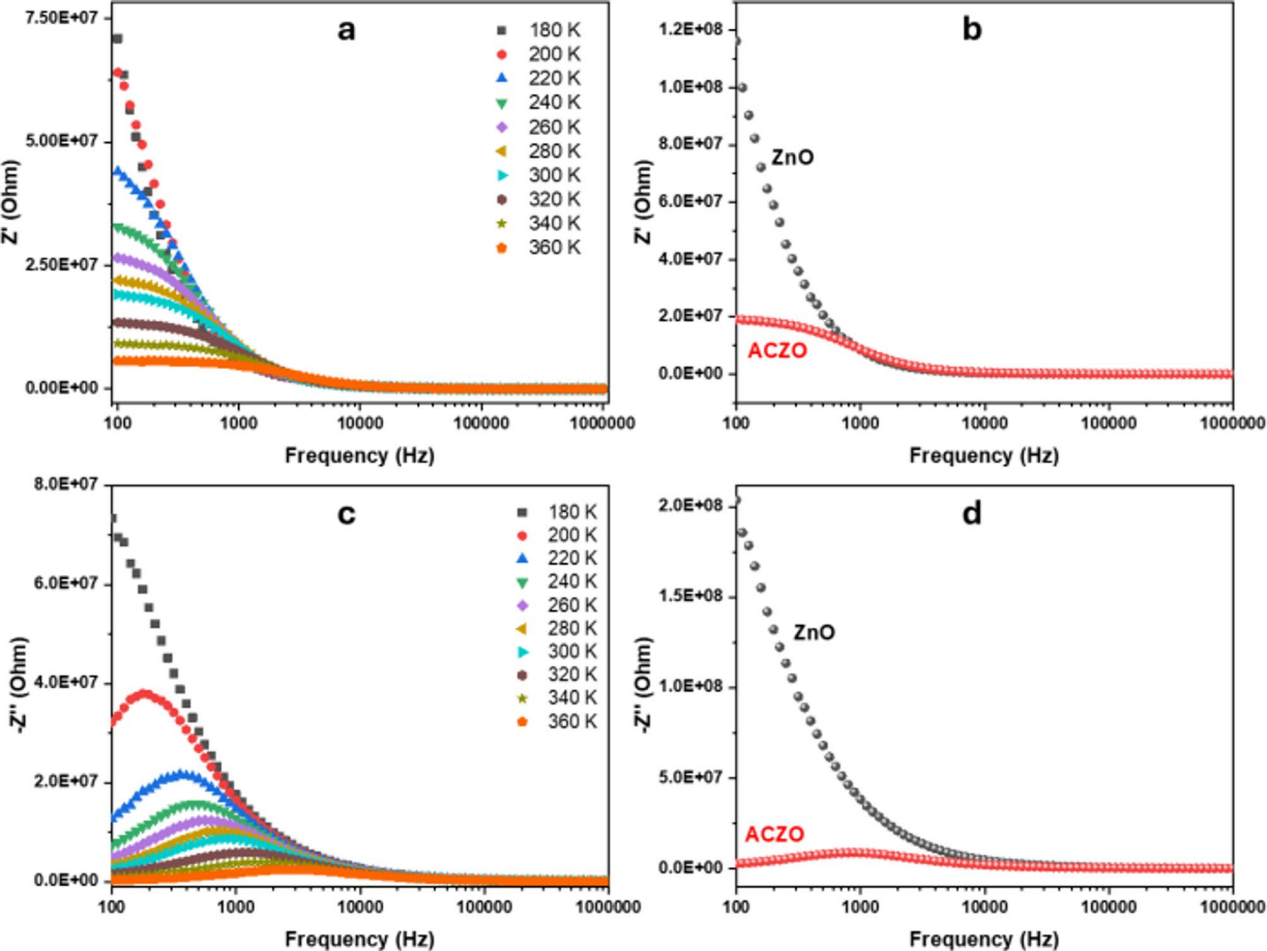
(a) Z′ vs. frequency for ACZO at different temperatures, (b) comparison of Z′ for ZnO and ACZO at 300 K, (c) Z″ vs. frequency for ACZO at 300 K, and (d) comparison of Z″ for ZnO and ACZO at 300 K.

Figure 6-a shows the real part of impedance (Z′) for the ACZO sample at different temperatures shows high values at low frequency and decreases progressively with increasing frequency. This behaviour indicates that grain boundaries and space charge polarization dominate at low frequencies, while the grains control the response at high frequencies^34,35^. The decrease of Z′ with increasing temperature confirms a thermally activated conduction process and improved charge carrier mobility in the co-doped material. Figure 6-b shows the comparison of Z′ between pure ZnO and ACZO at 300 K. It is clearly observed that the ACZO sample has lower impedance values over the whole frequency range. This means that Al–Ca co-doping reduces the resistive barriers and enhances electrical conductivity, most likely by increasing the free carrier concentration and improving grain boundary transport.

Figure 6-c shows The imaginary part of impedance (Z″) for the ACZO sample at 300 K presents a relaxation peak. The presence of a broad peak indicates a non-Debye type relaxation with a distribution of relaxation times, which is typical for nanostructured and defect-rich materials. The peak position reflects the characteristic relaxation process of charge carriers in the co-doped ZnO structure. Figure 6-d presents the comparison of Z″ between pure ZnO and ACZO at 300 K. The ACZO sample shows a lower intensity peak and a slight shift toward higher frequency compared to ZnO. This indicates reduced energy loss and faster relaxation dynamics in the co-doped sample, confirming that Al–Ca co-doping improves the electrical response and facilitates charge carrier movement.

**Fig. 7:**
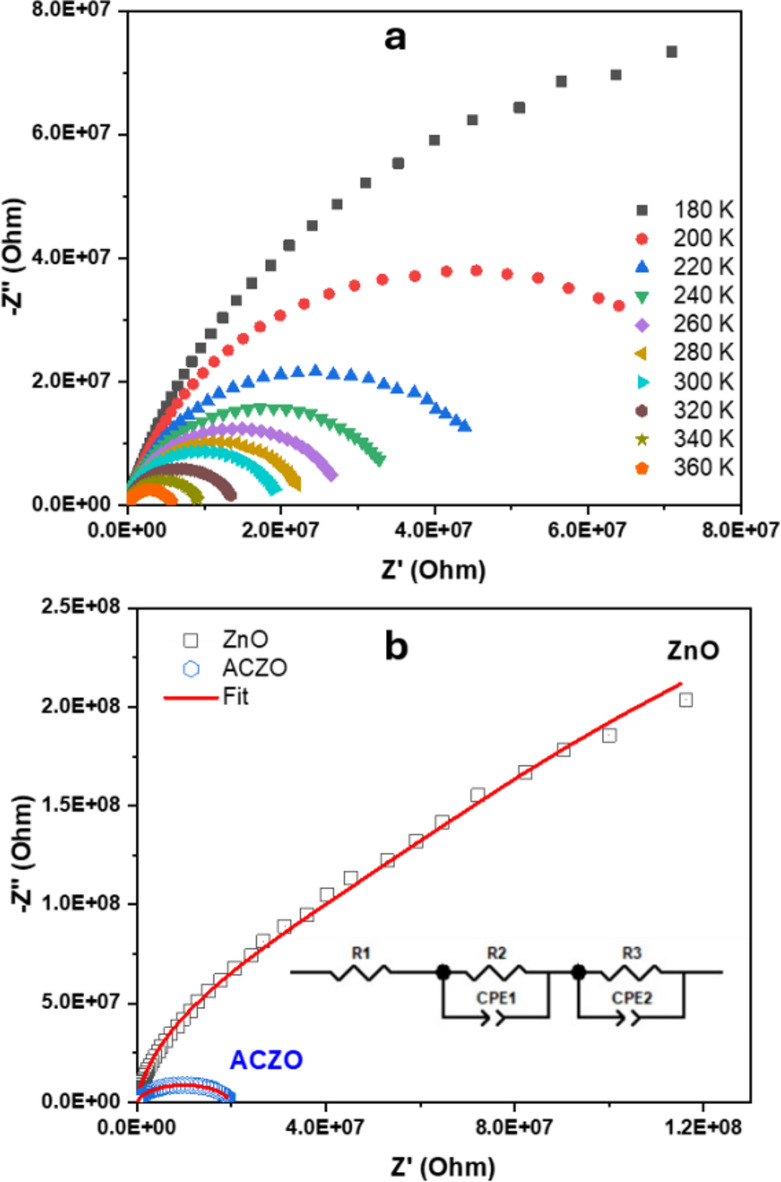
Nyquist diagram of (**a**) the ACZO under different temperature and (**b**) the ZnO and ACZO at 300 K.

The Nyquist plots of the complex impedance provide further insight into the electrical transport mechanisms of the samples. Figure 7-a presents the Nyquist diagram of the ACZO sample at different temperatures. The spectra exhibit depressed semicircular arcs whose centers are located below the real axis, indicating a non-Debye type relaxation behaviour associated with a distribution of relaxation times^36,37^. Such depressed arcs are commonly observed in doped ZnO ceramics and are generally attributed to structural disorder, defect states, and inhomogeneous charge carrier dynamics induced by dopant incorporation^38,39^. The presence of a single dominant semicircle suggests that the electrical response is mainly governed by grain boundary resistance, while the absence of a well-resolved second arc indicates that grain contributions are comparatively less pronounced within the measured frequency range. As the temperature increases, the diameter of the semicircle decreases progressively, which reflects a reduction in the bulk resistance and an enhancement of the electrical conductivity. This behaviour confirms the thermally activated nature of the conduction process and indicates that charge carriers gain sufficient energy to overcome the potential barriers at grain boundaries in the ACZO material. This trend is consistent with previous impedance studies on Al-, Ca-, and co-doped ZnO systems, where doping modifies grain boundary potential barriers, enhances carrier mobility, and consequently improves electrical transport properties^40,41^.

Figure 7-b shows the Nyquist plot at 300 K for both pure ZnO and ACZO samples, allowing a direct comparison between the undoped and co-doped systems. It is clearly observed that the semicircle corresponding to the ACZO sample has a much smaller diameter than that of pure ZnO, indicating a significant decrease in the total resistance after Al–Ca co-doping. To quantitatively support this observation, the experimental impedance data were fitted using the equivalent circuit (R₁ + (R₂//CPE₁) + (R₃//CPE₂)) shown in the inset of Fig. 7-b, and the extracted fitting parameters are summarized in Table 1. This reduction in resistance can be attributed to the increase in free carrier concentration introduced by Al³⁺ ions and to the modification of the defect structure and grain boundary potential barriers induced by Ca²⁺ ions. The fitting results reveal a pronounced decrease in both grain and grain-boundary resistances for the ACZO sample compared to pure ZnO, confirming that the co-doping effectively lowers the charge transport barriers. The use of constant phase elements (CPE₁ and CPE₂), with α values close to unity, indicates non-ideal capacitive behavior associated with electrical heterogeneity and distributed relaxation processes within the ceramic matrix. These secondary phases may also contribute to additional grain-boundary resistance and non-ideal capacitive behavior. Consequently, the co-doped ACZO sample exhibits improved charge transport and enhanced electrical conductivity compared to pure ZnO. These results are fully consistent with the conductivity and impedance analyses, confirming that Al–Ca co-doping is an effective approach to improve the electrical performance of ZnO-based materials.

**Table 1:** Equivalent circuit fitting parameters obtained from impedance spectroscopy for pure ZnO and Al–Ca codoped ZnO (ACZO) ceramics.

Sample	*R*_1_ (Ω)	*R*_2_ (Ω)	*R*_3_ (Ω)	CPE_1_ (F)	α_1_	CPE_2_ (F)	α_2_
**ZnO**	874.1	8.4344 × 10⁷	6.146 × 10⁸	1.363 × 10⁻¹¹	0.91537	6.118 × 10⁻¹²	1.044
**ACZO**	1241	195,160	1.949 × 10⁷	1.412 × 10⁻¹⁰	0.88207	1.810 × 10⁻¹¹	0.92596

In order to obtain a deeper understanding of the electrical response of the prepared materials, the dielectric properties, electric modulus formalism, and relaxation time behaviour were investigated. The study of the dielectric constant allows us to evaluate the polarization mechanisms and their dependence on frequency and temperature, while the electric modulus helps to suppress electrode effects and highlight the bulk relaxation processes. In addition, the analysis of relaxation time provides useful information about the charge carrier dynamics and transport mechanisms in the material. Together, these complementary approaches give a more complete view of the conduction and relaxation phenomena in the ZnO and ACZO systems.

In **Fig. ****S1****-a**, the dielectric constant of the ACZO sample decreases strongly with increasing frequency at all temperatures. The high values at low frequency are due to space charge accumulation at grain boundaries, consistent with Maxwell–Wagner interfacial polarization, while at high frequency the charge carriers cannot follow the field and the permittivity becomes almost constant. In **Fig. ****S1****-b**, a similar dispersion is observed, with values decreasing as frequency increases and slightly increasing with temperature, indicating that the polarization processes are thermally activated and involve both dipolar and interfacial contributions. In **Fig. ****S1****-c**, the same general trend is observed, with higher loss at low frequency followed by a gradual decrease toward high frequency; this behaviour reflects charge carrier hopping and grain boundary effects, which are typical for semiconducting oxide materials^42,43^.

The weak CaO and γ-Al₂O₃ secondary phases detected by XRD may slightly contribute to the dielectric response through interfacial polarization. Since these phases possess electrical properties different from ZnO, their interfaces with the ZnO matrix can promote localized charge accumulation under an alternating electric field, particularly at low frequencies. However, the very low intensity of their diffraction peaks indicates a limited volume fraction, suggesting that their contribution remains minor. Therefore, the dominant dielectric behavior is mainly governed by the Al–Ca co-doped ZnO matrix, while the secondary phases provide only a small contribution through grain-boundary and interfacial polarization effects.

**Fig. 8:**
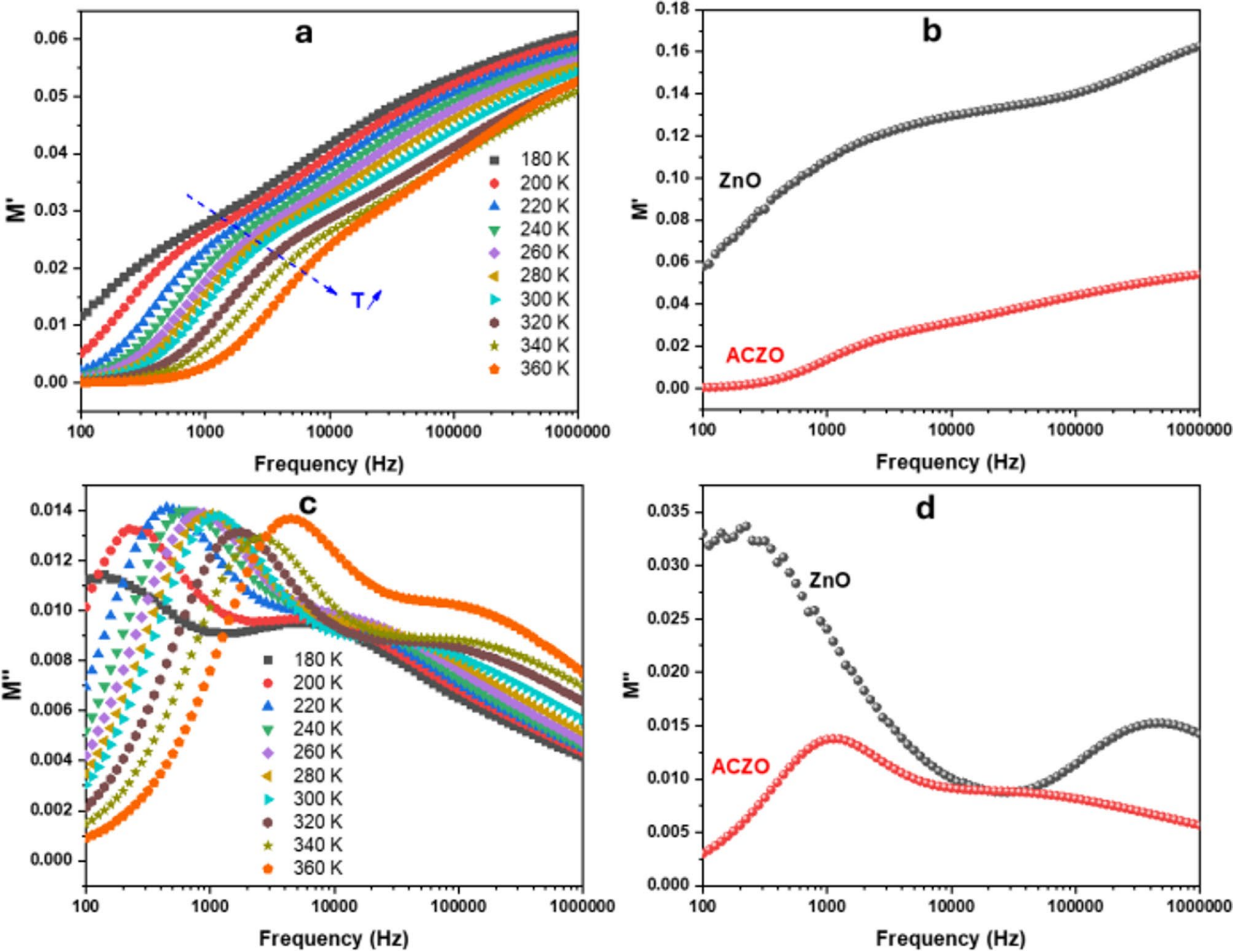
Electrical modulus spectra of the samples: (**a**) real part M′ of ACZO at different temperatures, (**b**) comparison of M′ for ZnO and ACZO at 300 K, (**c**) imaginary part M″ of ACZO at different temperatures, and (**d**) comparison of M″ for ZnO and ACZO at 300 K.

Figure 8 presents the electrical modulus analysis of the samples. In Fig. 8-a, the real part of the electric modulus (M′) of ACZO shows a gradual increase with frequency at all temperatures, starting from very low values at low frequency and tending to a saturation region at high frequency. This behaviour indicates that the electrode polarization is suppressed and that the charge carriers become more mobile with increasing frequency, reflecting a typical relaxation process in semiconducting oxides^44,45^. In Fig. 8-b, the comparison of M′ at 300 K between ZnO and ACZO clearly shows that the ACZO sample exhibits lower M′ values over the whole frequency range. This suggests that the co-doping with Al and Ca enhances the electrical conductivity and reduces the capacitive contribution, which is consistent with an increase in free charge carrier density in the ACZO sample compared to pure ZnO.

In Fig. 8-c, the imaginary part of the modulus (M″) for ACZO exhibits a well-defined relaxation peak that shifts toward higher frequencies with increasing temperature. This shift confirms a thermally activated relaxation process and indicates a decrease in relaxation time, which can be attributed to a hopping conduction mechanism of localized charge carriers^46,47^. Finally, in Fig. 8-d, the comparison of M″ at 300 K between ZnO and ACZO shows that the relaxation peak of ACZO appears at higher frequency and with lower intensity than that of ZnO. This behaviour indicates faster relaxation dynamics and lower resistance in the ACZO sample, confirming that Al and Ca co-doping improves the electrical transport properties and facilitates charge carrier mobility.

**Fig. 9:**
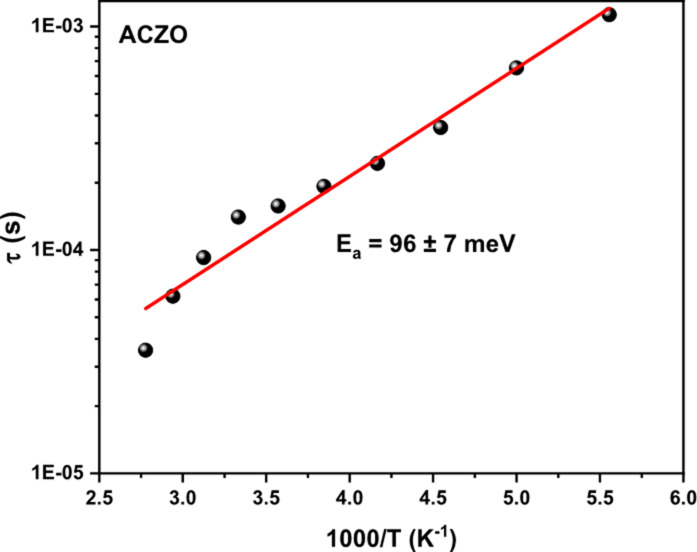
Semilogarithmic plot of the relaxation time versus 1000/T.

From the variation of the imaginary part of the electrical modulus (Mʺ) with frequency, the relaxation process of the ACZO sample can be clearly observed through the presence of well-defined peaks that shift toward higher frequencies when the temperature increases. For each temperature, the peak frequency (fₘₐₓ) was determined, and the corresponding relaxation time was estimated using the relation τ = 1/(2πfₘₐₓ)^48,49^. Then, by plotting ln(τ) as a function of 1000/T (Fig. 9), we obtained a straight line that follows an Arrhenius-type behavior, which allowed us to extract the activation energy of the relaxation process from the slope (E_a_=96 ± 5 meV). This activation energy represents the energy barrier that the charge carriers need to overcome to contribute to the hopping conduction mechanism in the material.

When we compare this activation energy with the one obtained from the DC electrical conductivity (112 ± 5 meV), we find that the two values are very close. This agreement suggests that both the relaxation process and the electrical conduction originate from the same physical mechanism, which is most likely the thermally activated hopping of localized charge carriers, such as electrons associated with defects or oxygen vacancies, across the grains and grain boundaries. This consistency between modulus analysis and conductivity analysis gives a good confidence in the conduction model of the ACZO sample and confirms that the charge transport is dominated by a thermally activated process.

## Conclusion

In summary, Al–Ca co-doped ZnO nanopowders were successfully prepared and their structural, morphological, and electrical properties were carefully investigated. The co-doping preserves the wurtzite ZnO structure while reducing the crystallite size and introducing a controlled amount of lattice distortion and defects. These structural modifications play an important role in enhancing the electrical and dielectric properties of the material. The ACZO sample shows higher conductivity and lower impedance compared to pure ZnO, which confirms that Al³⁺ increases free carrier concentration while Ca²⁺ modifies the defect structure and grain boundary barriers. The electrical transport follows a thermally activated hopping mechanism, as confirmed by both Arrhenius conductivity analysis and electrical modulus relaxation study, which provide very similar activation energies. The dielectric and impedance results also indicate a non-Debye type relaxation and strong contribution of grain boundaries and space charge polarization. Although trace amounts of CaO and γ-Al₂O₃ secondary phases were detected, their contribution is mainly associated with grain-boundary and interfacial polarization effects, while the dominant electrical and dielectric response originates from the co-doped ZnO matrix. CaO and γ-Al₂O₃ mainly act as interfacial modifiers, while the ACZO matrix remains the dominant contributor to electrical behavior. Overall, the combination of Al and Ca doping offers a simple and effective way to improve the electrical performance and stability of ZnO. This makes ACZO a promising material for applications in transparent electronics, sensors, and other functional devices where enhanced charge transport and dielectric response are required.

## Supplementary Information

Below is the link to the electronic supplementary material.

Supplementary Material 1

## Data Availability

Data are available from the corresponding author upon reasonable request.
