# Supplementary material for: Al–Ca co-doped ZnO nanostructures for enhanced electrical and dielectric properties
Source: Sci Rep. 2026 Jul 24;16:25701. doi: 10.1038/s41598-026-61523-5 (PMC13482645; doi:10.1038/s41598-026-61523-5)
Supplement: Supplementary file 1 [file 41598_2026_61523_MOESM1_ESM.docx]

**Suppliment Material**

**Advanced multifunctional ZnO nanoparticles via Al–Ca co-doping for enhanced electrical and dielectric applications**

**Nouf Ahmed Althumairi ^1^, Imen Hammami ^2^, Sonia Soltani ^3^, R. A. El-Kasaby ^4, 5^, Mokhtar Hjiri ^6^, Majdi Benamara ^2,*^, S. Soreto Teixeira ^2^, M.P.F. Graça ^2^, Manuel Almeida Valente ^2^**

*^1^ Department of Physics, College of Science, Majmaah University, Al-Majmaah 11952, Saudi Arabia*

*^2^ i3N and Physics Department, University of Aveiro, Campus Universitário de Santiago, Aveiro 3810-193, Portugal*

*^3^ Department of Physics, College of Science, Qassim University, Buraidah, 51452, Saudi Arabia*

*^4^ Department of Chemistry, College of Science, Taibah University, Al-Madinah Al-Munawarah 30002, Saudi Arabia*

*^5^ Chemistry Department, Women's College for Arts Science and Education, Ain Shams University, Cairo 11566, Egypt*

*^6^ Department of Physics, College of Sciences, Imam Mohammad Ibn Saud Islamic University (IMSIU), Riyadh 11623, Saudi Arabia*

*^*^Correspondance :* [*majdi.benamara@ua.pt*](mailto:majdi.benamara@ua.pt)

**Figure S1.** Frequency dependence of (**a**) real dielectric part (ε′), (**b**) imaginary dielectric part (ε″), and (**c**) dielectric loss (tan δ) of the ACZO sample at different temperatures.
